# Accuracy of Predictive Formulas vs. Indirect Calorimetry in Estimating Energy Needs of Patients in Intensive Care Units

**DOI:** 10.3390/healthcare14091139

**Published:** 2026-04-24

**Authors:** Didem Aybike Haspolat, Aslı Gizem Çapar, Şule Göktürk

**Affiliations:** 1 Nutrition and Dietetics Department, Nuh Naci Yazgan University, Kayseri 38170, Türkiye; gizem_pekmezci@hotmail.com; 2 Department of Neurosurgery, Kayseri City Hospital, Kayseri 38080, Türkiye; sulegokturk@outlook.com

**Keywords:** Bland–Atman analysis, ESPEN, Harris–Benedict formulas, intensive care unit patient, indirect calorimeter

## Abstract

**Highlights:**

**What are the main findings?**

- Indirect calorimetry and predictive formulas (Harris–Benedict equation and European Society for Clinical Nutrition and Metabolism (ESPEN) recommendations) show limited agreement in estimating the energy requirements of mechanically ventilated patients in intensive care units.
- The energy intake of patients in intensive care units was frequently lower than those estimated by both predictive methods, emphasizing variability in metabolic needs.

**What are the implications of the main findings?**

- Individualized monitoring of energy expenditure, particularly using indirect calorimetry when available, may improve the accuracy of nutritional support in critically ill patients.
- Relying solely on predictive formulas may lead to overestimation of energy needs, highlighting the need for careful clinical assessment to optimize outcomes.

**Abstract:**

**Introduction**: Accurately meeting the energy requirements of patients in intensive care units (ICUs) is crucial to prevent catabolism, muscle loss, and complications. We assessed their energy needs in this study using indirect calorimetry (IC) and predictive formulas, comparing the results with delivered energy intake and evaluating agreement. **Materials and Methods**: A total of 38 mechanically ventilated patients in seven ICUs at Kayseri City Hospital were included; eligible patients were ≥18 years old and mechanically ventilated for at least 24 h. Disease severity and nutritional risk were evaluated using validated indices (prognostic nutritional index (PNI) and Modified Nutrition Risk in the Critically Ill (mNUTRIC)), and basal energy expenditure (BEE) was measured by IC and calculated using the Harris–Benedict (HB) and ESPEN formulas. IC measurements lasted 15 min under resting conditions in conscious patients and, according to acute phase criteria, in unconscious patients in a quiet, temperature-controlled environment. Nutrition was provided enterally or parenterally based on patient condition and disease severity. Agreement between IC and predictive formulas was assessed using Bland–Altman analysis, a statistical method that evaluates agreement between two measurement techniques. **Results**: Estimated energy requirements differed significantly from delivered energy intake (*p* < 0.001). IC-derived values were significantly lower than those estimated by the HB equation and ESPEN recommendations (*p* < 0.001), suggesting that predictive equations may overestimate energy requirements in this population. By contrast, delivered energy intake was lower than IC-measured values, with a mean difference of approximately 503 kcal, indicating a potential risk of underfeeding in clinical practice. Weak correlations were observed between methods (IC vs. HB: r = 0.35, *p* = 0.003; IC vs. ESPEN: r = −0.21, *p* = 0.02), indicating limited agreement between predictive equations and IC measurements, and Passing–Bablok regression analysis further supported this lack of agreement between methods. **Conclusions**: The energy intake delivered to patients was lower than the calculated values. Indirect calorimetry is important for accurately monitoring and determining energy requirements based on delivered energy intake, and further research in this area is needed. These findings highlight the importance of individualized monitoring of energy expenditure in critically ill patients and suggest that reliance solely on predictive equations may lead to clinically relevant discrepancies in energy delivery.

## 1. Introduction

Providing adequate energy to critically ill patients during and after intensive care remains a fundamental challenge in clinical nutrition. Although this topic has been widely addressed in the literature, important uncertainties persist regarding the agreement between estimated energy requirements and the energy intake delivered to patients in clinical practice [1,2,3]. Gastrointestinal intolerance and impaired nutrient absorption may hinder patients from achieving prescribed energy targets, potentially leading to malnutrition [4]. In addition, assessing nutritional status in critically ill patients is inherently complex due to factors such as sedation, loss of consciousness, mechanical ventilation, and the use of enteral or parenteral nutrition. Furthermore, commonly used laboratory parameters (particularly albumin) and their long half-lives may not accurately reflect current nutritional status, thereby complicating the evaluation of energy intake adequacy. Evidence on energy and protein intake during and after critical illness remains limited and heterogeneous. A systematic review highlighted substantial variability in energy intake relative to estimated requirements and emphasized the limited number of high-quality studies evaluating nutritional adequacy beyond the acute intensive care unit (ICU) phase [5]. In addition, recent reviews have demonstrated that energy and protein intake in critically ill patients often remain inconsistent with target requirements and that important gaps persist in current evidence [6,7]. For example, the mean delivered energy intake in critically ill patients was reported in a prospective study to only reach approximately 58% of the calculated requirements, while protein intake remained around 45% of target values [8]. Similarly, in a study evaluating 81 adult ICU patients followed for ≥48 h, only 10% of 60 patients achieved the recommended protein intake of 1.2 g/kg/day, and 28% met the recommended energy intake of 25 kcal/kg/day at 12 months [9]. Both under- and overfeeding are associated with adverse clinical outcomes. Inadequate energy intake may result in weight loss, muscle wasting, impaired immune function, prolonged recovery, and increased mortality, whereas excessive energy provision may lead to hyperglycemia, metabolic complications, and organ dysfunction [2,10]. Therefore, accurate estimation and appropriate delivery of energy are essential for optimizing patient outcomes. To guide nutritional therapy, energy requirements are commonly estimated using indirect calorimetry (IC) or predictive equations [8,11]. Although more than 200 equations have been developed to estimate basal energy expenditure (BEE), their accuracy is limited due to inter-individual variability in metabolic responses [12,13]. Indirect calorimetry, by measuring oxygen consumption (VO_2_) and carbon dioxide production (VCO_2_), allows a more individualized assessment of energy expenditure and substrate utilization through the respiratory quotient (RQ) [3,14]. Despite being considered the gold standard, its use in clinical practice remains limited due to cost, technical constraints, and patient-related factors [1,2,4].

Although previous studies have examined energy requirements and nutritional adequacy in critically ill patients, few have directly evaluated the agreement between the calculated energy requirements and the delivered energy intake provided through enteral or parenteral nutrition in the hospital setting. Moreover, most existing studies have primarily focused on estimated or prescribed energy targets rather than the energy delivered in clinical practice. In addition, studies simultaneously comparing indirect calorimetry and predictive equations with delivered energy intake within the same patient population remain limited. Therefore, the extent to which commonly used estimation methods reflect real-world nutritional delivery remains unclear, particularly in ICU settings. More specifically, existing studies suggest that discrepancies between energy requirements measured by indirect calorimetry (IC) and the energy actually delivered to patients may be common across different clinical settings. In mechanically ventilated critically ill patients, energy intake may exceed measured energy expenditure by approximately 500 kcal/day, particularly when non-intentional energy sources such as propofol and dextrose are considered [15]. By contrast, in patients with minor burns, energy intake may remain insufficient, covering only about 71% of the measured energy expenditure, indicating a potential risk of underfeeding despite increased metabolic demands [16]. Similarly, studies on surgical patients receiving parenteral nutrition have reported inconsistencies between IC-measured energy expenditure and predictive equations, suggesting that energy requirements may vary over time and may not be fully captured by static estimation methods [17]. Furthermore, predictive equations such as Harris–Benedict have shown limited agreement with IC measurements, with acceptable accuracy observed in only about half of patients and variability influenced by clinical factors such as inflammation and metabolic stress [18]. In combination, these results suggest that both over- and underfeeding may remain clinically relevant risks, and that commonly used estimation methods may not always accurately reflect true energy requirements across different patient populations. Although the literature on energy requirements in critically ill patients has expanded in recent years, the relationship between estimated energy needs and the energy actually delivered in clinical practice remains unclear. Most studies have focused either on estimating energy expenditure or on predefined nutritional targets, while relatively few have examined how these estimates translate into real-world energy delivery at the bedside. In particular, studies simultaneously comparing indirect calorimetry-derived energy requirements, predictive equations, and delivered energy intake within the same patient population are limited. Therefore, it remains uncertain to what extent these methods reflect routine clinical nutrition practice in intensive care settings. In this study, we compare energy requirements estimated by indirect calorimetry and widely used predictive equations (Harris–Benedict, ESPEN) with delivered energy intake in critically ill patients in the intensive care units of a hospital in Turkey.

This study was previously presented as a conference abstract, reporting the initial findings, at the 2024 ESPEN Congress held in Milan, Italy [19].

## 2. Materials and Methods

### 2.1. Study Area

This cross-sectional observational study was conducted with 38 mechanically ventilated patients who were hospitalized in a total of seven ICUs (General Surgery, Pulmonology, Anesthesiology, Neurology, Coronary, Internal Medicine, and Cardiology) at Kayseri City Hospital (KCH). During the study period (May–June 2023), consecutive patient enrollment was applied.

### 2.2. Study Plan and Sample Selection

**Figure 1:**
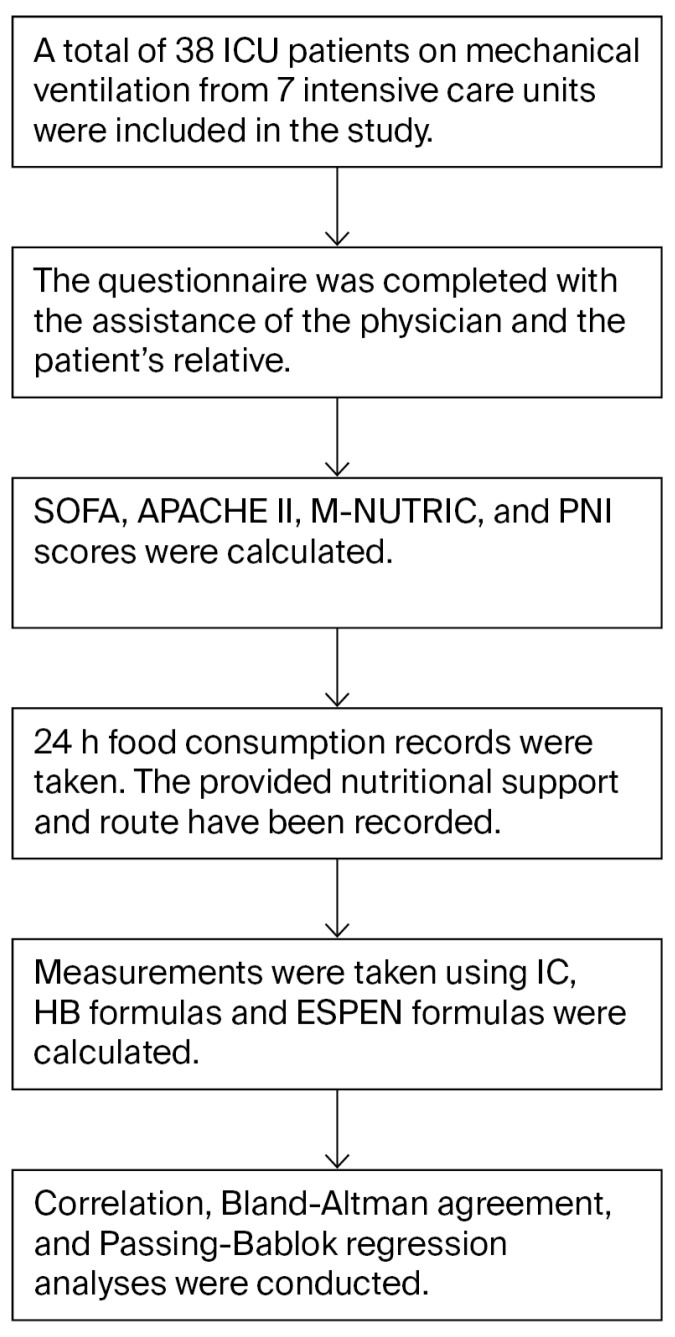
Flow chart of patient selection and study procedures.

Patients were recruited using a consecutive sampling approach during the study period. The daily BEE requirements calculated during hospitalization and the energy intake by the patients were determined, which were then compared with the values calculated using Indirect Calorimetry (COSMED, Rome, Italy) IC and different formulas in an attempt to ascertain the value that was closest to the delivered energy intake. The disease severity and nutritional risk of the patients were assessed using the prognostic nutritional index (PNI), sequential organ failure assessment (SOFA), acute physiology and chronic health evaluation II (APACHE II), and modified nutritional risk in critically ill score (mNUTRIC) (Figure 1) Additionally, biochemical findings and anthropometric measurements were determined. The sample size calculation was based on the difference in mean BEE values reported by Ana Caroline da Silva Oliveira et al. [20]. An effect size of 0.48, power of 80% (1 − β = 0.8), and alpha level of 0.05 were applied, and following reviewers’ suggestion, an estimated effect size of 0.48 was also calculated and added to the study. It was thus determined that 36 patients would be sufficient for an adequate sample size.

This study received ethical approval from the Nuh Naci Yazgan University (NNYU) Scientific Research and Publication Ethics Committee under protocol number 2022/003-007 dated 10 November 2022, and was conducted in accordance with the principles of the Declaration of Helsinki. Institutional permission was obtained from KCH with decision number 76397871/799.

Patients were included in the study if they were intubated or undergoing mechanical ventilation via tracheostomy in the last 24 h or longer, aged 18 and above, and showed no significant changes in mechanical ventilation parameters (FiO_2_, airway pressure, respiratory rate, minute ventilation) during the periods when IC measurements were taken. Fraction of inspired oxygen (FiO_2_) was recorded during mechanical ventilation. Patients were excluded if they required FiO_2_ > 0.6 mmHg and PEEP ≥ 20 cm/H_2_O, were hyperthermic (>38 °C) or hypothermic (<35 °C), had amputated extremities, suffered air leaks in any of the respiratory circuits, had an RQ ratio outside the physiological limits (0.7–1.2) non-compliant with mechanical ventilation, or had any condition that could affect the measurement.

### 2.3. Data Collection

The data in this study were collected by the researchers using patient files and information obtained in collaboration with physicians and the relatives of patients. In cases where information was missing or required clarification, the relatives were contacted to ensure data completeness and consistency.

A 34-item structured questionnaire (data collection form), developed by the researchers, was used to collect the patients’ sociodemographic characteristics, anthropometric measurements, and ICU-related clinical information. The form included sections on demographic characteristics (3 items), anthropometric measurements (3 items), chronic diseases and ICU-related conditions (9 items), and nutritional status (11 items). Additionally, detailed information on the nutrition, laboratory findings, and clinical status of the patients was obtained from ICU nurse monitoring forms. Enteral and parenteral nutrition plans prescribed by physicians, nutrition products used, and administration routes were recorded.

### 2.4. Determining Malnutrition Risk

#### 2.4.1. Modified Nutritional Risk in Critically Ill (mNUTRIC) Assessment

The mNUTRIC score is the first validated nutritional risk assessment tool for use with critically ill patients [21]. It is calculated (without interleukin-6 values) for patients at nutritional risk by using their age, number of comorbid diseases, days from hospital admission to ICU admission, and APACHE II and SOFA scores. Patients with scores ≥5 are categorized as having ‘high’ mortality and high malnutrition risk, while ≤4 are classified as having ‘low’ mortality and low malnutrition risk [22].

#### 2.4.2. Prognostic Nutritional İndex (PNI) Assessment

The PNI score was used for prognostically evaluating the nutritional status and disease process of the patients. Patients with scores below the median are considered to have a high mortality rate and classified as having high malnutrition [23,24].

Calculation and evaluation of the PNI score:

PNI = (10 × Albumin/dL) + (0.005 × Lymphocyte count (mm^3^).

PNI < 43.7: High mortality and high malnutrition.

PNI ≥ 43.7 and ≤51.4: Moderate mortality and moderate malnutrition.

PNI > 51.4: Considered a low mortality rate and low malnutrition.

### 2.5. Nutrition Therapy Plan

The researchers did not provide any nutritional treatment interventions or recommendations, and the documentation included all details of the enteral or parenteral nutrition plan implemented by the physician; the enteral and parenteral products utilized; the preferred method of nutrition; and admission, treatment, and onset of nutrition during hospitalization. Enteral products were administered via a nasogastric tube, percutaneous endoscopic gastrostomy, jejunostomy, and the nasoduodenal route; parenteral products were administered through a central catheter route. Records of a three-day nutrition plan were obtained, which consisted of the day of and the two days preceding IC measurement. The energy and nutrient contents of the enteral and parenteral products were taken from the information on the label, and the daily intake amounts were calculated using Microsoft Excel 2016. In this study, “energy intake” refers to the energy actually delivered to patients in clinical practice, rather than prescribed or theoretically calculated values. Delivered energy intake is defined as the amount of energy actually delivered to patients via enteral or parenteral nutrition. The nutritional plans implemented in the hospital were obtained from the patient follow-up form, and the BEE requirement was calculated using a formula based on the guidelines of the European Society for Clinical Nutrition and Metabolism (ESPEN) [1]. Those receiving less than 60% of the measured energy (targeted energy) by IC were considered hypocaloric, between 60 and 100% were considered normocaloric, and more than 100% were considered hypercaloric [1,25]. While ready-made liquid solutions are preferred via a catheter for total parenteral nutrition, ready-made standard commercial products (1 mL/1 kcal) were used for enteral nutrition via enteral nutrition routes. Furthermore, nutrition below <70% of the targeted energy is expressed as ‘insufficient nutrition’, and above >110% is expressed as ‘excessive nutrition’ [1].

Clinical variables potentially acting as confounders, including disease severity, sepsis status, ventilatory support, hemodynamic instability, and feeding tolerance, were extracted from detailed patient records and assessed by an intensivist involved in ICU management. This ensured a clinically informed evaluation of patient heterogeneity. In addition, a standardized enteral nutrition protocol was applied across all intensive care units, starting at 10 mL/h with stepwise escalation according to tolerance. No feeding interruptions were observed during the study period, which helped minimize variability in energy delivery related to clinical practice.

### 2.6. Calculating Basal Energy Expenditure

#### 2.6.1. Indirect Calorimetry Protocol

The BEE of patients was measured by a physician and a researcher using an indirect calorimeter (COSMED, Q-NRG+, Rome, Italy) for 15 min [26,27]. During the measurement, the patient was instructed to remain at rest, motionless, and lying down, while the environment was kept quiet and at a constant room temperature. Pneumotach Flowmeter-Flow REE: A Pneumotach Flowmeter is a device that measures air flow and is commonly used in respiratory studies, pulmonary function tests, or to monitor ventilation systems. The device has a flow range of 0–1.6 L/s, flow accuracy of ±2% or ±20 mL/s, ventilation range of 1–25 L/min, and ventilation accuracy of ±2% or ±100 mL/min [28].

#### 2.6.2. Formula Protocol

In the article ‘The Centenary of the Harris-Benedict Equations: How to Best Assess Energy Requirements’ published in 2021, the ESPEN Expert Group’s recommendations state that the Harris–Benedict (HB) equation is still one of the best predictive formulas for patients who require nutritional interventions. We thus chose this equation for this study.

The BEE of patients who were in the ICU was calculated according to the recommended 25–30 kcal per kilogram by ESPEN and the HB formula, shown below [29].

Harris–Benedict Formula:

Male: BEE (kcal/day) = 66.47 + (13.75 × weight in kg) + (5.003 × height in cm) − (6.755 × age in years).

Female: BEE (kcal/day) = 655.1 + (9.563 × weight in kg) + (1.85 × height in cm) − (4.676 × age in years).

### 2.7. Biochemical Findings

The biochemical findings of the patients on the measurement day were obtained from digital patient records stored in the Kayseri City Hospital’s Hospital Information Management System. Serum potassium, sodium, leukocyte, lymphocyte, albumin, hematocrit, BUN, ALT, AST, neutrophil, platelet, CRP, calcium, glucose, PO_2_, PCO_2_, HCO_3_, FiO_2_, O_2_H_b_, and SO_2_ levels were examined and interpreted according to the reference values of Kayseri City Hospital [30].

### 2.8. Anthropometric Measurements

The body weights of sedentary and bedridden patients were measured by nurses using Schroder brand (Klingenberg am Main, Germany) SCH 4040 T weighing bedwith a calibration of 0.1 kg. The formula ‘height = 153.492 − (7.97 × gender [gender: male = 1, female = 2]) + (0.974 × ulna length (UL) cm)’ was used to determine the height [31], and the body weight (kg) of patients divided by their height (m^2^) was used to calculate BMI (kg/m^2^). According to the World Health Organization (WHO) adult classification, individuals with a BMI of <18.5 kg/m^2^ are classified as being underweight, 18.5–24.9 kg/m^2^ are classified as being of normal weight, 25.0–29.9 kg/m^2^ are classified as being overweight, and 30.0 kg/m^2^ and above are classified as being obese [32].

### 2.9. Statistical Analysis

The analysis of the data obtained in this study was performed using SPSS Statistics 26 and the TURCOSA program. The descriptive statistics are presented in number of units (*n*), percentage (%), mean ± standard deviation ($\bar{x}$ ± ss), median (M), 25th percentile (Q1), and 75th percentile (Q3) values. Normally distributed quantitative data is presented as mean ± standard deviation ($\bar{x}$ ± ss), while non-normally distributed quantitative data is given as median (25–75). In group comparisons, Student’s *t*-test and Kruskal–Wallis test were used for quantitative data, and Friedman test and Nemenyi post hoc analysis were applied to compare the three groups. Correlation analysis was used to assess the relationship between variables, while agreement between methods was evaluated using Bland–Altman analysis and Passing–Bablok regression. A significance level of *p* < 0.05 was considered.

## 3. Results

### 3.1. Participant Characteristics

Of the patients included in this study, 63.15% (*n* = 24) were male and 36.84% (*n* = 14) were female, and among these patients, 60.52% (*n* = 23) had a chronic disease. A total of 52.70% (*n* = 20) were receiving enteral nutrition via a nasogastric (NG) tube (47.37%) and percutaneous endoscopic gastrostomy (PEG) (5.26%), while 47.37% were receiving nutrition via the intravenous route. Of the patients studied, 47.36% (*n* = 18) had a normal BMI, while all patients had a prognostic nutritional index (PNI) score < 43.7, indicating malnutrition. According to ESPEN criteria, 52.63% of the patients were classified as malnourished (Table 1).

### 3.2. Energy Requirements and Nutritional Status

When indirect calorimetry (IC) measurements were taken as a reference, 42.10% of the participants were hypocaloric and 34.20% were hypercaloric. The energy requirement estimated using the Harris–Benedict (HB) formula [1685.50 (1526.00–1883.75) kcal] and calculated according to ESPEN recommendations (1889.84 ± 327.55 kcal) was significantly higher than that measured by IC [1470.00 (1243.00–1848.25) kcal] (*p* < 0.001) (Table 2).

The patients’ serum sodium, albumin, hematocrit, O_2_Hb (%), PO_2_ (mmHg), calcium (mg/L), and SO_2_ (%) values were below the reference range, whereas neutrophil count, BUN, HCO_3_, CRP, and glucose levels were above it. There were similarities in energy and nutrient intakes between patients classified as malnourished according to the PNI and those with an mNUTRIC score ≥ 5 (Table 3).

### 3.3. Correlation and Agreement Analyses

**Figure 2:**
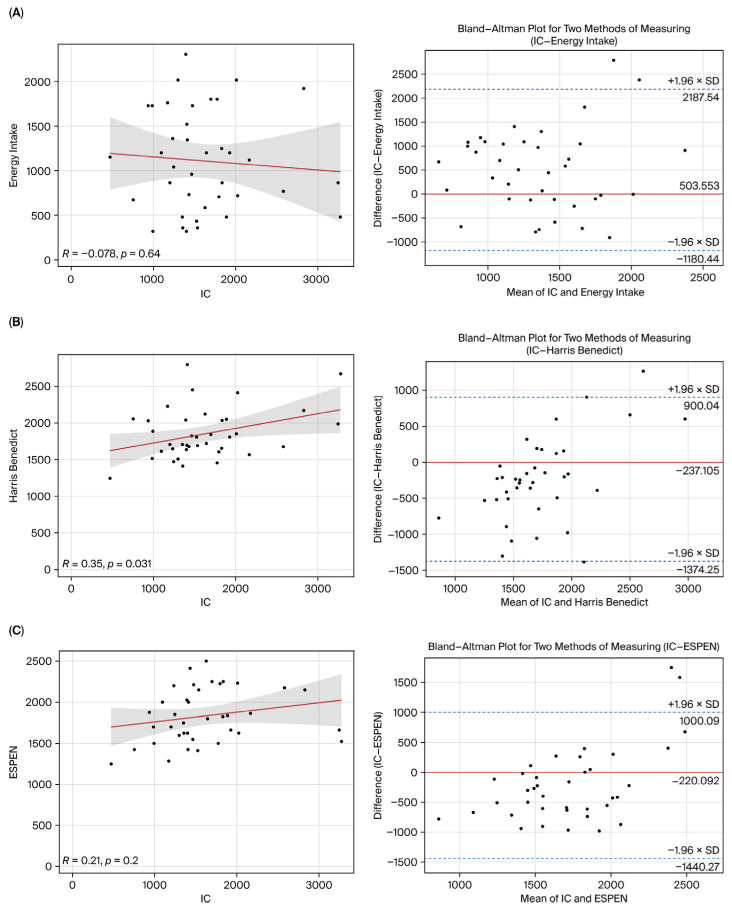
The correlation and agreement between different measurements for basal energy expenditure and the reference indirect calorimetry (IC) measurement. Comparisons included energy intake (**A**), Harris–Benedict equation (**B**), and ESPEN recommendations (**C**). Scatter plots with Pearson correlation analysis. The red line represents the linear regression curve, and the shaded area indicates the 95% confidence interval. Bland–Altman analysis. The solid horizontal line represents the mean difference (bias), while the dashed lines indicate the upper and lower limits of agreement (95%).

There was a weak positive correlation between IC and HB (r = 0.35, *p* = 0.003) and a weak negative correlation between IC and ESPEN (r = −0.21, *p* = 0.02). The basal energy requirement determined by IC was 237.105 kcal lower than that of the HB formula and 220.192 kcal lower than that of the ESPEN recommendations according to Bland–Altman analysis (*p* < 0.001). The value measured by IC was 503.553 kcal higher than the delivered energy intake by the patients. Passing–Bablok regression analysis demonstrated both systematic and proportional differences among the IC, HB, and ESPEN methods. In addition, the low concordance correlation coefficients indicated poor agreement between these methods. These findings suggest that the HB and ESPEN methods do not measure energy requirements in a manner comparable to IC and that the level of agreement between methods is limited. Hospital nutrition intake did not differ significantly from other methods (*p* > 0.05) (Table 4, Figure 2).

## 4. Discussion

One of the main findings of our research is the discrepancy between the estimated energy requirements, based on the HB equation and ESPEN recommendations, and the energy delivered to patients in clinical practice when their basal metabolic rate (BMR) measured by IC is used as a reference (Figure 2). It has been observed that the estimated energy requirements calculated using frequently recommended energy requirement formulas (ESPEN: 1812.50 kcal (1586.75–2156.25), HB: 1765.00 kcal (1630.75–2042.00)) are higher than those based on IC measurement results (1470.00 kcal (1243.00–1848.25)). Furthermore, it has been found that the daily energy intake provided to patients in clinical practice (1080.00 kcal (650.25–1728.00)) is lower than that measured by IC (Table 4).

The finding that the delivered energy intake was on average 503.553 kcal lower than IC-measured requirements may be considered noteworthy, and this discrepancy may be more closely related to clinical practice rather than the limitations of the calculation methods. Previous studies have demonstrated that predictive equations show variable accuracy and may not reliably reflect true energy expenditure in critically ill patients [33]. In addition, indirect calorimetry has been reported to provide a more accurate assessment of energy requirements compared to predictive methods [34,35]. Furthermore, recent evidence suggests that indirect calorimetry-guided nutrition may improve energy balance and may be associated with better clinical outcomes [36]. Therefore, the energy gap observed in the present study may be attributable to real-world clinical nutrition practices rather than solely to inaccuracies in predictive equations. Although we demonstrate in our study significant discrepancies between estimated and measured energy requirements, the absence of clinical outcome data such as mortality, length of hospital stay, and complications limits the interpretation of the clinical significance of these findings. Therefore, while these discrepancies may potentially contribute to risks such as under- or overfeeding, their actual impact on patient outcomes cannot be determined within the scope of this study. This is particularly relevant in critically ill patients, where even relatively small deviations in energy delivery may influence metabolic balance and overall clinical course. However, this potential relationship cannot be confirmed in the present study.

These findings suggest that the observed differences between methods should be interpreted cautiously in clinical practice, as they may reflect both the methodological limitations of predictive equations and variability in real-world nutritional management. Given the complexity of metabolic responses in critically ill patients, individualized assessment of energy requirements may be more appropriate than reliance on fixed predictive formulas alone. This situation indicates that estimated energy requirement formulas may overestimate the delivered energy needs of patients, and providing more energy than necessary to critically ill patients may increase metabolic burden. However, our results have shown that the energy requirements of patients are often not met, increasing the risk of malnutrition (52.63%), which is one of the greatest threats in clinical practice. These findings suggest that the discrepancy between estimated, measured, and delivered energy may reflect both the methodological limitations of predictive equations and real-world clinical practices. We therefore suggest that indirect calorimetry (IC) may be useful for a more accurate assessment of BMR and that meeting daily energy requirements based on these measurements may positively impact the critical care process in ICUs. Although indirect calorimetry is considered the reference method for assessing energy expenditure in critically ill patients, its routine clinical use is limited by cost, feasibility, and accessibility constraints. These limitations restrict its widespread implementation in everyday intensive care practice despite its methodological advantages. For this reason, its use is often confined to selected patients or research settings in many ICUs [1,37,38].

Initiating nutrition support, achieving nutritional targets, and maintaining sustainability are crucial for the prognosis of patients in the ICU. It is recognized that accurately determining the energy consumption based on metabolic requirements alone is insufficient for these patients. It is imperative to ensure that patients are neither under- nor overfed to prevent complications [39]. According to ASPEN and ESPEN guidelines, the weakness of estimation-based equations and the use of IC have been the subjects of various evaluations and recommendations. Both methods are preferred to assess the energy needs of patients in the ICU [40].

In the studies conducted, EE in ICU patients under mechanical ventilation was compared using IC and commonly used predictive equations such as HB, Schofield, Swinamer, Penn State, and Ireton-Jones. The results showed that while all equations correlated with IC measurements, there were variations in accuracy across different patient subgroups. However, the presence of correlation does not necessarily indicate agreement between methods. It was noted that the predictive equations showed systematic bias, and using IC may be useful when treating critically ill patients to avoid under- or overestimating their metabolic needs [41,42].

The main finding of this study was a consistent discrepancy between estimated energy requirements and delivered energy intake in critically ill patients, suggesting frequent underfeeding in clinical practice. The most important finding of our study is that the targets estimated by both indirect calorimetry (IC) and predictive equations were not met, suggesting that patients are underfed rather than overfed (42.10% hypocaloric, 34.20% hypercaloric; Table 1). It is important to note that delivered energy intake in this study was calculated as the mean of three consecutive days, including the measurement day. The patients included in this period were those who tolerated feeding and in whom nutritional intake had been gradually advanced toward target levels; therefore, the analysis mainly reflects patients with more stable feeding tolerance rather than those in the very early phase of low-dose feeding. Although ICU nutritional protocols often involve gradual advancement of feeding, using a three-day average reduces day-to-day fluctuations in energy delivery. However, inter-patient differences in feeding tolerance and variability in the rate of advancement toward target nutrition may still influence the mean energy intake [43]. This apparent discrepancy between overestimation by predictive equations and underfeeding in clinical practice may be explained by feeding interruptions, gastrointestinal intolerance, hemodynamic instability, and conservative feeding strategies, particularly during the early phase of critical illness. Therefore, these discrepancies cannot be attributed solely to the accuracy of predictive equations, but should also be interpreted in light of the multidimensional clinical factors influencing the amount of energy actually delivered. Furthermore, the involvement of dietitians or nutrition specialists in ICU teams may improve the accuracy of nutritional planning, enhance monitoring of energy delivery, and support timely adjustments in response to clinical changes. Additionally, the Passing–Bablok regression analysis revealed that although a relationship was found between energy requirement values calculated by IC and formulas, they were incompatible with one another.

The appropriate energy requirement for critically ill patients remains a subject of debate. While patients at high nutritional risk may benefit from early, full-calorie feeding, early aggressive feeding in patients at low nutritional risk may increase morbidity and mortality [1].

In critical conditions such as sepsis and trauma, there is an increase in energy requirements in the first five days following injury, accompanied by a decrease in structural protein levels and an increase in acute-phase proteins. Considering that this catabolic state leads to a significant reduction in lean body mass [8], the importance of meeting energy requirements becomes evident. However, when comparing the daily intake of patients with IC-based values in this study, a substantial proportion of patients were inadequately nourished (median intake: 1080.00 kcal vs. IC: 1470.00 kcal; Table 4). These findings indicate that inadequate energy intake may be accompanied by impaired nutritional status in critically ill patients. This comparison has an inherent conceptual limitation, as delivered energy intake in critically ill patients is influenced by multiple clinical factors, including feeding interruptions, gastrointestinal intolerance, hemodynamic instability, and stepwise nutritional protocols, which limit direct comparability with estimated energy requirements.

Furthermore, analysis of three-day dietary intake showed that both total energy and macronutrient intake remained below recommended levels (energy fulfillment: 60.06%; protein fulfillment: 46.47%; Table 2), suggesting not only insufficient but also imbalanced nutrient intake. The standardized stepwise feeding protocol applied in the ICU (10 cc, 20 cc, 30 cc progression) may have contributed to the inability to achieve calculated energy targets. This standardized approach may represent an important clinical factor underlying the observed energy deficit. Inadequate nutrition leading to malnutrition can result in multiple adverse outcomes, including impaired respiratory function and delayed recovery [1]. These effects may be mediated by increased protein catabolism, loss of lean body mass, and impaired immune response.

Studies have shown that the HB equation significantly underestimates EE compared to IC, while ESPEN guidelines overestimate EE [15,20]. Similarly, in our study, ESPEN-based values were higher than IC measurements (1889.84 ± 327.55 vs. 1470.00 kcal; Table 2). Although a positive correlation was observed between IC and HB (r = 0.35, *p* = 0.003), and a negative correlation was identified between IC and ESPEN (r = −0.21, *p* = 0.02) (Table 4), correlation alone is not sufficient in demonstrating agreement between methods. Therefore, agreement was evaluated using Bland–Altman analysis, which showed a lack of concordance (Table 5, Figure 2). This finding may indicate that relying solely on predictive equations without considering energy delivery could lead to clinically relevant discrepancies. Passing–Bablok analysis further confirmed the lack of agreement between methods (Table 5).

This study has several limitations. Although the sample size was considered adequate based on power analysis, it was conducted as part of a master’s thesis and was subject to time and budget constraints, which may have limited the statistical power of the study. While all eligible patients from the hospital’s intensive care unit were included during the study period, the relatively small sample size (*n* = 38), mainly due to the strict inclusion criteria and the observational design of the study, may have reduced the statistical power. This may have limited the ability to fully capture the clinical and metabolic heterogeneity of critically ill patients, potentially affecting the robustness of subgroup analyses.

In addition, although the study was conducted across all intensive care units within the same hospital, the single-center design may still reflect local clinical practices and patient characteristics. Therefore, the findings may not be fully generalizable to other institutions and healthcare settings. Only two predictive equations were used despite the availability of numerous methods for estimating energy expenditure, and measurements were performed within the first 24–48 h of ICU admission, when metabolic responses are highly dynamic. Patient heterogeneity may have influenced the findings despite the applied exclusion criteria. In addition, comparisons between estimated and delivered energy may have been affected by gastrointestinal tolerance, feeding interruptions, and clinical decision making, introducing potential bias. The single-center design and lack of clinical outcome data may limit generalizability. Finally, although indirect calorimetry is considered the reference method, its clinical applicability is limited by cost, accessibility, and methodological constraints, and the absence of longitudinal follow-up restricts the evaluation of long-term outcomes. The observed differences between estimated energy requirements, indirect calorimetry measurements, and delivered energy in critically ill patients should be interpreted with caution in the context of clinical variability and study limitations. These findings should be considered within the framework of real-world intensive care practice, where variability in disease severity, physiological status, and metabolic responses may influence energy balance. Although potential confounding factors were carefully assessed and a standardized feeding protocol was applied, complete elimination of clinical variability is not feasible in critically ill patients. Therefore, the observed discrepancies between estimated, measured, and delivered energy may partly reflect the inherent heterogeneity of ICU practice as well as physiological differences among patients.

## 5. Conclusions

In conclusion, there is a clinically relevant discrepancy between estimated energy requirements, indirect calorimetry measurements, and delivered energy in critically ill patients, with a tendency toward underfeeding in clinical practice. Indirect calorimetry is considered a reference method for energy assessment; however, its routine use is limited by cost, feasibility, and accessibility constraints. When IC is not available, predictive equations may be used as alternatives, but careful monitoring of delivered energy remains essential in ICU practice.

## Figures and Tables

**Table 1:** Demographic, Clinical, and Nutritional Characteristics of ICU Patients (*n* = 38).

Variables	Categories	*n*	%
Gender	Male	24	63.15%
	Female	14	36.84%
Chronic illness	Yes	23	60.52%
	No	15	39.47%
Body Mass Index (BMI)	<18.5	2	5.26%
	18.5–24.9	18	47.36%
	25.0–29.9	11	28.94%
	>30	7	18.42%
Nutrition status	Undernourished	38	100.00%
Nutrition pattern	Enteral nutrition	20	52.70%
	Total parenteral nutrition	18	47.30%
Nutritional pathway	NG	18	47.37%
	PEG	2	5.26%
	IV	18	47.37%
Feeding regimen	Continuous infusion	32	84.21%
	Intermittent infusion	6	15.78%
Prognostic nutritional index score assessment (PNI)	<43.7	38	100.00%
Energy consumption according to IC measurement (kcal)	<60% Hypocaloric	16	42.10%
	60–100% Normocaloric	9	23.70%
	>100% Hypercaloric	13	34.20%
Nutrition status level	<70% Malnutrition	20	52.63%
	70–110% Adequate Nutrition	10	26.31%
	>110% Excessive Nutrition	8	21.05%

NG: Nasogastric feeding; PEG: Percutaneous endoscopic gastrostomy; IV: Intravenous.

**Table 2:** Clinical Scores and Macronutrient Intake of ICU Patients (*n* = 38).

Variable	Mean ± SD/Median (25–75%)
APACHE II score	22.50 (18.75–27.25)
BMI kg/m^2^	24.20 (21.72–28.22)
SOFA score	6.18 ± 2.608
m-Nutric score	4.71 ± 1.522
PNI score	31.17 ± 7.286
Energy (kcal)	1080.0 (650.25–1728.0)
Energy fulfillment (%)	60.06 (31.27–89.57)
Carbohydrate (g)	99.55 (64.33–195.90)
Carbohydrate fulfillment (%)	66.36 (42.87–153.60)
Protein (g)	44.54 (25.68–67.10)
Protein fulfillment (%)	46.47 (24.23–68.95)
Fat (g)	40.99 (21.69–59.43)
Fat fulfillment (%)	35.70 ± 22.212

If the data are normally distributed, Mean ± SD is used; if the data are not normally distributed, Median (25–75%) is used. APACHE II: Acute Physiology and Chronic Health Evaluation II; BMI: Body Mass Index; SOFA: Sepsis-related Organ Failure Assessment; m-Nutric: modified Nutrition Risk in Critically ill; PNI: Prognostic Nutritional Index.

**Table 3:** Comparison of Patients’ Daily Energy and Nutrient Intake According to PNI and m-Nutric Score Used in Determining Malnutrition Risk.

Variables	Group	Mean ± SD/Median (25–75%)	*p*
Energy (kcal)	Malnutrition: PNI < 43.7 (*n* = 38)	1080.0 (650.25–1728)	0.965
	High nutritional index: mNUTRIC ≥ 5 (*n* = 21)	1120.0 (532.5–1572.0)	
	Low nutritional index: mNUTRIC ≤ 4 (*n* = 17)	1040.0 (737.33–1728.00)	
Energy fulfillment (%)	Malnutrition: PNI < 43.7 (*n* = 38)	60.06 (31.27–89.57)	0.681
	High nutritional index: mNUTRIC ≥ 5 (*n* = 21)	60.13 (26.77–85.20)	
	Low nutritional index: mNUTRIC ≤ 4 (*n* = 17)	56.21 (33.52–90.73)	
Carbohydrate (g)	Malnutrition: PNI < 43.7 (*n* = 38)	99.55 (64.33–195.90)	0.961
	High nutritional index: mNUTRIC ≥ 5 (*n* = 21)	96.00 (59.86–172.80)	
	Low nutritional index: mNUTRIC ≤ 4 (*n* = 17)	103.10 (67.37–230.40)	
Carbohydrate (%)	Malnutrition: PNI < 43.7 (*n* = 38)	53.33 (29.84–53.70)	0.894
	High nutritional index: mNUTRIC ≥ 5 (*n* = 21)	53.33 (29.83–53.51)	
	Low nutritional index: mNUTRIC ≤ 4 (*n* = 17)	53.33 (31.29–53.70)	
Protein (g)	Malnutrition: PNI < 43.7 (*n* = 38)	44.54 (25.68–67.10)	0.801
	High nutritional index mNUTRIC ≥ 5 (*n* = 21)	44.35 (21.77–64.95)	
	Low nutritional index: mNUTRIC ≤ 4 (*n* = 17)	52.00 (31.98–67.10)	
Protein (%)	Malnutrition: PNI < 43.7 (*n* = 38)	15.84 (15.53–19.90)	0.984
	High nutritional index: mNUTRIC ≥ 5 (*n* = 21)	15.84 (15.53–19.94)	
	Low nutritional index: mNUTRIC ≤ 4 (*n* = 17)	15.84 (15.53–18.99)	
Fat (g)	Malnutrition: PNI < 43.7 (*n* = 38)	40.99 (21.65–59.43)	0.861
	High nutritional index: mNUTRIC ≥ 5 (*n* = 21)	38.40 (19.38–60.00)	
	Low nutritional index: mNUTRIC ≤ 4 (*n* = 17)	46.89 (27.32–58.42)	
Fat (%)	Malnutrition: PNI < 43.7(*n* = 38)	30.28 (30.00–48.87)	0.868
	High nutritional index: mNUTRIC ≥ 5 (*n* = 21)	30.28 (30.00–48.89)	
	Low nutritional index: mNUTRIC ≤ 4 (*n* = 17)	30.29 (30.00–48.90)	

Data are not normally distributed, Median (25–75%) is used. For *p* value tested using a Kruskal–Wallis. m-Nutric: modified Nutrition Risk in Critically ill; PNI: Prognostic Nutritional Index.

**Table 4:** Energy Requirements of Intensive Care Patients Obtained by Different.

	Group	Median (25–75%)	*p*
Energy consumption	IC (kcal)	1470.00 (1243.00–1848.25) ^a^	<0.001
	HB (kcal)	1765.00 (1630.75–2042.00) ^b^	
	ESPEN (kcal)	1812.50 (1586.75–2156.25) ^b^	
	Hospital nutrition intake (kcal)	1080.00 (650.25–1728.00)	

Data are not normally distributed, Median (25–75%) is used. Friedman test with Nemenyi post hoc analysis was used a < b, *p* < 0.05. Values with different letters (a, b) indicate statistically significant differences between groups (*p* < 0.05).

**Table 5:** Comparison and agreement statistics between Energy Requirements of Intensive Care Patients Obtained by Different Methods and Their Comparison.

Parameter Estimates	Passing–Bablok Regression		Agreement Statistics	
	*β*_0_	*β*_1_	ICC	CCC
Energy intake-IC				
Coefficient	−794.74	1.17	−0.17	−0.06
95% CI	−3598.41–297.56	0.48–3.02	−1.25–0.39	−0.29–0.18
Interpretation	No systematic error	No proportional error	No agreement	No agreement
Harris Benedict-IC				
Coefficient	1076.26	0.45	0.46	0.27
95% CI	662.16–1329.52	0.26–0.74	−0.03–0.72	0.03–0.48
Interpretation	Yes systematic error	Yes proportional error	No agreement	No agreement
ESPEN-IC				
Coefficient	998.52	0.51	0.30	0.16
95% CI	476.76–1283.81	0.31–0.90	−0.34–0.64	−0.08–0.39
Interpretation	Yes systematic error	Yes proportional error	No agreement	No agreement

The comparison between the three energy prediction methods and indirect calorimetry was performed using Passing–Bablok regression to assess systematic and proportional errors, and ICC and CCC to evaluate agreement and consistency between measurements. ICC: Intra-class correlation coefficient; CCC: Concordance correlation coefficient; CI: Confidence interval; IC: Indirect Calorimetry; ESPEN: The European Society of Parenteral and Enteral Nutrition.

## Data Availability

The data presented in this study are available on request from the corresponding author.

## Abbreviations

The following abbreviations are used in this manuscript:

ICU	Intensive Care Unit
IC	Indirect Calorimetry
HB	Harris–Benedict
ESPEN	European Society for Clinical Nutrition and Metabolism
BEE	Basal Energy Expenditure
BMR	Basal Metabolic Rate
EE	Energy Expenditure
PNI	Prognostic Nutritional Index
SOFA	Sequential Organ Failure Assessment
APACHE II	Acute Physiology and Chronic Health Evaluation II
mNUTRIC	Modified Nutrition Risk in Critically Ill
RQ	Respiratory Quotient
VO_2_	Oxygen Consumption
VCO_2_	Carbon Dioxide Production
FiO_2_	Fraction of Inspired Oxygen
PEEP	Positive End-Expiratory Pressure
BMI	Body Mass Index
WHO	World Health Organization
ASPEN	American Society for Parenteral and Enteral Nutrition
NG	Nasogastric (tube)
PEG	Percutaneous Endoscopic Gastrostomy
CRP	C-Reactive Protein
BUN	Blood Urea Nitrogen
ALT	Alanine Aminotransferase
AST	Aspartate Aminotransferase
O_2_Hb	Oxyhemoglobin
PO_2_	Partial Pressure of Oxygen
PCO_2_	Partial Pressure of Carbon Dioxide
HCO_3_^−^	Bicarbonate
SO_2_	Oxygen Saturation
REE	Resting Energy Expenditure
UL	Ulna Length
